# Not just spontaneous remission: Time-dependent and independent effects in pre-intervention symptom reduction

**DOI:** 10.1016/j.invent.2026.100926

**Published:** 2026-02-27

**Authors:** Vilgot Huhn, Nils Hentati Isacsson, Marie Bendix, Martin Kraepelien, Hanna Sahlin, Viktor Kaldo, Erik Forsell

**Affiliations:** a Centre for Psychiatry Research, Department of Clinical Neuroscience, Karolinska Institutet, Stockholm Health Care Services, Region Stockholm, Sweden; b Umeå University, Department of Clinical Sciences/Psychiatry, Umeå, Sweden; c Department of Psychology, Faculty of Health and Life Sciences, Linnaeus University, Växjö, Sweden

**Keywords:** Regression towards the mean, Social anxiety disorder, Panic disorder, Depression, Health anxiety, Insomnia, Natural course, Symptom fluctuations, Measurement reactivity

## Abstract

Psychological symptoms tend to change over time, even in the absence of clinical intervention. For example, self-ratings are often higher at screening compared to start of treatment. A plausible hypothesis is that this is due to patients' self-referring when their gradually fluctuating symptoms are worse than usual. That hypothesis predicts that patients that wait longer will have had longer time to return to their average symptom level. On the other hand, other processes related to measurement reactivity, contact with a clinician, or regression towards the mean, do not predict a time-dependent relationship.

Our aim was to estimate the extent of this hypothesized symptom reduction in depression, social anxiety disorder, panic disorder, health anxiety and insomnia (both total reduction and the relationship with time). The sample included adults (*N* = 8744) from an outpatient psychiatric clinic providing ICBT in Swedish routine care. Time-dependent effects were estimated with linear regression for both primary symptoms and secondary depressive symptoms. A simulation of symptom fluctuations was built to estimate power and further contextualize the effects.

Patients improved on average from screening to the start of the intervention, but this varied substantially depending on diagnosis and questionnaire used. The waiting time weakly predicted the degree of improvement both for primary depressive symptoms and comorbid depressive symptoms. The estimate for primary depressive symptoms was sensitive to modeling choices, shrinking towards zero when modeled with fat-tailed residuals. The preponderance of “immediate” reductions in symptoms have implications for reporting standards of pre-treatment-measurements, especially in single-group intervention studies.

## Highlights

• Self-rated symptoms tend to improve between screening and start of treatment.
• Waiting time only weakly predicts symptom improvement when waiting for ICBT treatments.
• The relationship with waiting time is most clear with comorbid depressive symptoms.
• Most symptom reduction is “immediate” or independent of waiting time.
• Timing and measurement context must be clearly reported to optimize interpretability.

## 1 Introduction

Why do patients get better? In psychotherapy research and clinical practice, many patients show a symptomatic improvement that occurs during the time period between identification (e.g. screening or diagnostic assessment) and treatment start, (Barkham et al., 2006; Bisby et al., 2023; Hansen et al., 2002; Hostmaelingen et al., 2023; Howard et al., 1986; Krogsboll et al., 2009; Steinert et al., 2017). These improvements somehow occur before the actual therapeutic content of the treatment have had any chance to affect symptoms.

Broadly, there have been many suggested explanations for improvements that are not caused by treatments, e.g. spontaneous improvement, common factors, and statistical artifacts (Eysenck, 1994; Subotnik, 1972). For example, in the context of studies with passive control groups, a meta-analysis including 37 clinical trials and 2900 patients that covered 8 clinical conditions, Krogsboll et al. (2009) attributed 35% of improvement in depression severity to spontaneous recovery, and a further 24% to placebo effects. However, whether the same processes are at work in the apparent improvement *before* treatment even starts is unknown.

The overall causes for pre-treatment symptom change can be divided in two broad categories; (1) causes or processes related to the passing of time, and (2) immediate, or very time-limited, processess unrelated to how much time elapses. In the present study we will refer to these as time-dependent contra time-independent effects, but in most cases the crucial difference is in how quickly a process unfolds (days or moments).

### 1.1 Time dependent effects

While “regression towards the mean” is sometimes used as a catch-all term for all spuriously identified within-group changes where extreme measurements get less extreme over time, some consider this use of the term imprecise (Maraun et al., 2011). There are numerous processes that can theoretically be related how symptoms change through the passage of time. A non-exhaustive and partially conceptually overlapping list includes: (a) The overall concept of spontaneous recovery or natural course (Cuijpers, 2018). Many psychological disorders naturally wax and wane (Merikangas et al., 2003; Wittchen et al., 2000), being partially episodic while also strongly related to stable traits like neuroticism (Brown, 2007). (b) Functional behavior changes, coping or problem solving triggered when symptoms reaches a certain threshold (Hagger et al., 2017). This could be either self-directed or by ones surrounding reacting. The act of seeking health-care can plausibly be accompanied by other helpful behaviors such as reaching out to one's support network or taking sick-leave. (c) External causes of symptoms that come and go; Symptoms of depression, anxiety and insomnia have been consistently linked to current stressors and negative life events (Kendler et al., 1998; Yang et al., 2018). (d) Furthermore, internal and external fluctuations, such as seasonal changes (Zhang and Volkow, 2023), or endocrine fluctuations (Walker 2nd et al., 2020) have been found to be associated with psychological symptoms.

**Fig. 1:**
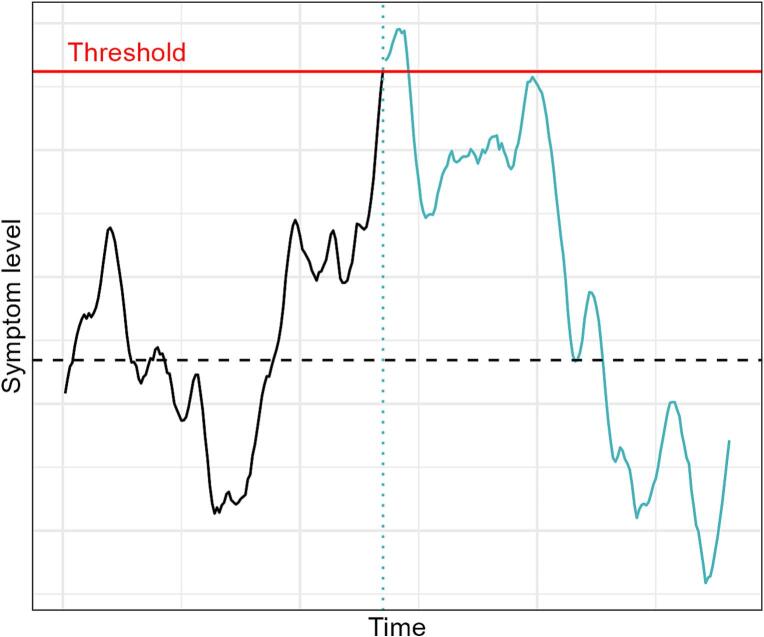
Schematic representation of symptom fluctuations. Note. Example of fluctuating symptoms for one patient. When symptoms reach some critical level (red line), the patient is motivated to seek healthcare. After that the patient become identified for the clinic (blue dotted line). Symptoms then continue to fluctuate and tend to return back towards the individual mean symptom level (dashed black line). Blue color indicates the time period that is potentially visible for the clinic, after the patient has sought help.

For our purposes the common theme among these is that symptoms fluctuate over time for various reasons, and that individuals are hypothesized to be more likely to seek treatment when their symptoms are worse than usual. Thus, at the point when patients get to the clinic, they are more likely to improve than deteriorate (see Fig. 1 below for an imagined example of one patient's symptom trajectory).

### 1.2 Time independent effects

Reasons for symptom change theoretically unrelated to the passage of time can be divided in two broad categories; (1) Reactions to measurement or to contact with clinicians, or (2) Regression towards the mean (i.e. a statistical artefact).

(1) *Reactions To Measurment Or To Contact With Clinicians.*

Various sources of assessment reactivity are a recurrent methodological problem with self-ratings, i.e. when the act of measurement or the surrounding context affects the rating. For example, Johar and Sackett (2018) found that repeated measurements of negative emotions in an experimental setting tend to become less negative and hypothesized this was because of increased attention to emotion from the measurement event itself.

In a clinical setting exaggerated screening scores compared to pre-treatment scores could occur if patients report more severe or intense symptoms at screening in order to elicit help from the mental health professionals, thus consciously or unconsciously amplifying symptoms (Arrindell, 2001). Sarwar et al. (2022) describes how patient symptom exaggeration could be used as an attempt to persuade the clinician to see their own viewpoint, due to lack of better communication skills or trust in the assessing clinician. It could also be due to the self-report screening measures increasing patient awareness of symptoms and thereby an initial overreporting of symptoms, leading to inflated scores compared to later self-ratings (i.e. pre-treatment) (Capellan et al., 2017). Furthermore, self-report measures themselves may inadvertently signal that higher symptoms need to be endorsed in order to get help (Merckelbach et al., 2019). Additionally, social desirability to indicate gratefulness for having received the treatment could affect ratings (Arrindell, 2001). Finally, emotional reactions to the questionnaire, for example being triggered by words and phrases or being nervous for misunderstanding the questions or providing the wrong answers, could be more pronounced the first time the questionnaire is answered and understanding of concepts could evolve over time, altering the answers (Arrindell, 2001).

It has also been suggested that even before an intervention has started, the assessment process may induce “placebo effects” or contain therapeutic “common factors” (Cuijpers, 2018). Suggested factors have been: Therapeutic effect of contact with clinician and/or relief, better understanding of symptoms, deriving ideas of how to cope from questions asked, positive expectations and attention experienced after finally seeking help for your troubles (Constantino et al., 2011; Frank and Frank, 1991; Mojtabai, 2005). Furthermore, a single assessment visit can also be seen as a health intervention as it combines a structured psychopathologic assessment with elements of person-centered listening, psychoeducation, counseling and care planning. Thus, the assessment visit per se may have a real clinical impact (Clarke et al., 2019). Single visits have for example been found to be effective in changing alcohol habits (Kaner et al., 2018; O'Donnell et al., 2014) and single psychoeducational sessions may decrease depressive symptoms (Ghafoori et al., 2016). Further, even healthcare contacts without the aim of therapeutic intervention where patients only report their behavior may lead to behavior change (Godin et al., 2008; McCambridge and Kypri, 2011).

(2) *Regression Towards The Mean*

In trials where there is a cut-off for inclusion, and the basis for inclusion is measured with some measurement error, regression towards the mean will inflate the estimated within-group change (Morton and Torgerson, 2003). Since the cut-off is applied to the measured value, not the true value, patients that are actually above the threshold but due to measurement error gets a score below it are not included in the treatment, while patients that are truly below threshold but due to measurement error gets above are included. Thus the mean measured value of the sample will exceed the true mean. Upon re-measuring there is no selection threshold that biases the measured mean away from the true mean. As mentioned before, the uses of the term “regression towards the mean” varies (Maraun et al., 2011) and sometimes real, accurately measured, fluctuations regressing towards some individual long-term mean (or set-point) are included in the concept (e.g. Bland and Altman, 1994; Fitzmaurice, 2000). However, for our purposes the distinction between apparent fluctuation due to measurement error and real fluctuation in true scores is crucial. In the latter case symptoms reaching some critical level of underlying symptomatology is what causes the patient to seek treatment, while in the former case the score on the questionnaire is what permits the patient to be included in the trial.

### 1.3 The present study

This division into time-dependent and time-independent effects has potentially important interpretative implications in clinical evaluations. When investigating outcomes in real routine care settings it is common to not have a control group and thus conclusions are necessarily based on within-group change. For the sake of argument, let's imagine a clinic where patients tend to present with symptoms that are bound to spontaneously decrease over time. In that case, a pre-post comparison can potentially differ a lot depending on when “pre” symptoms are measured (e.g. at admission/screening, vs. after assessment but before treatment start, vs. right as treatment starts). While this problem no doubt is acknowledged by many researchers – often mentioned using the term regression towards the mean – the extent to which this change depends on time has rarely been investigated.

This division of time-dependent and time-independent effects is also potentially relevant for interpreting the literature. Sometimes reporting is unclear as to which measurement is defined as “pre” - whether it comes from a clinical assessment, a pre-assessment screening, or right at the start of treatment (e.g. Hobbs et al., 2017; Mewton et al., 2012; Titov et al., 2020). Thus, exploring if the change in symptoms when patients are waiting for psychological treatment tend to be dependent on time or not would be informative when interpreting the results of outcome assessments. Time-dependent explanations suggest that this pre-intervention change is only relevant to consider when some critical amount of time has elapsed between screening and start of treatment, whereas time-independent explanations (such as reactivity, help-seeking exaggerations, therapeutic effects of assessment) would suggest that this effect is important to take into account regardless of how much time passes between screening and treatment start.

The Internet Psychiatry Clinic in Stockholm, an outpatient clinic delivering internet-based, guided CBT treatment to self-referred patients from all of Sweden, has existed since 2007 and accumulated an appropriately large dataset to investigate this issue. At the clinic, how long a patient will wait before assessment will be largely dependent upon factors exogenous to the individual patients, for example how many other patients happen to seek treatment at that time, or if the clinic is temporarily understaffed. By taking advantage of these natural variations in waiting times between screening and treatment start we can estimate the relationship between symptom change and time. Patients that have waited for longer were predicted to have had more time to improve.

### 1.4 Aims

This study aims to replicate previous findings that patients on waiting lists tend to present with an average reduction in symptom scores and explore whether the hypothesized decrease is dependent on waiting time. In addition we aim to explore if this change differs between different diagnoses.

## 2 Method

### 2.1 Participants and procedure

The Internet Psychiatry Clinic (IPSY) in Stockholm, Sweden, provides internet delivered cognitive behavior treatment (ICBT) as guided self-help for various psychiatric disorders. Treatments are 9–13 weeks long and consist of text-based modules, homework assignments and an in-platform text messaging system where licensed psychologists asynchronously provide feedback, answer questions, and monitor progress. For further description of the clinic see Titov et al. (2018).

All participants in the current study received treatment at IPSY. Participants were recruited from all over Sweden via online self-referral, which includes filling out a series of screening questionnaires. The patient logs on to the national public healthcare platform with their electronic ID and immediately fill in their screening questionnaires. After the self-referral, all patients got an appointment for an assessment. In Sweden, there is an access to care-rule that dictates that a patient should get their first appointment after self-referral within 30 days, if at all possible. At IPSY, the majority of patients do get their first visit within that timeframe, though waiting periods fluctuate depending on various factors, and have sometimes been considerably longer. When treatment is started, patients are informed via SMS-messaging and upon first login fill out the pre-treatment questionnaires.

During the period 2007–2019, assessment visits took place face-to-face at the clinic, which is situated in the south of Stockholm. At the start of the Covid-19 pandemic, assessments were quickly shifted to be online only, via secure video conferencing services. From early 2020 and onward all assessments are via video.

Data from 2007 to 2020 is only for treatment of Major Depression (MDD), Social Anxiety Disorder (SAD) and Panic Disorder (PD). In January of 2021, the clinic shifted to a new technical platform for delivering online treatments. After this timepoint, data on treatment for Insomnia and Health Anxiety (HA) are added to the sample.

**Fig. 2:**
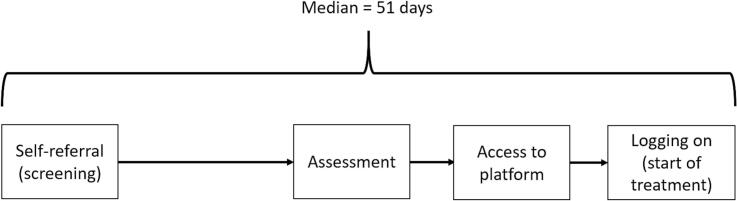
Timeline of events.

After assessment, there is little to no waiting time for treatment start. Patients wait only as long as it takes for administrators to process the inclusion and start treatment. This usually takes 0–2 days, and rarely as long as a week. When patients log on to the treatment platform for the first time, they fill out their first symptom ratings: this is our “start of treatment” ratings in the subsequent data-analysis (Fig. 2).

The current study follows patients from January 2008 to September 2024 from their screening at self-referral and up until the start of treatment. Patients must read the research participant information when they self-refer, and consent is obtained via an opt-out procedure in accordance with the ethical permission granted by the Swedish Research Ethics Authority (2011/2091–31/3 and 2018/2550–32). A total of 19 patients at IPSY have opted out and are not included in research.

The resulting sample available for analysis contained 8744 patients. See Table 2 for patients per treatment condition.

### 2.2 Measures

The primary outcome for this study is change over time between self-referral (screening) and the start of treatment (pre) for the primary symptom for which the patient receives treatment as well as for symptoms of depression as a secondary symptom (i.e. not in the MDD-treatment) depending on the analysis. The symptoms are assessed using widespread and reliable self-reported symptom questionnaires.

For depression the outcome measure was the Montgomery-Åsberg Depression Rating Scale Self-report version (MADRS-S) (Montgomery and Åsberg, 1979; Svanborg and Åsberg, 1994; Svanborg and Åsberg, 2001). MADRS-S has nine items, scores range from 0 to 54 points and the measure has high test-retest reliability (ICC = 0.78) (Fantino and Moore, 2009).

For social anxiety, the outcome measure was either the Leibowitz Social Anxiety Scale-Self report version (LSAS-SR) (Baker et al., 2002; Fresco et al., 2001) or SPIN (Connor et al., 2000). The LSAS has 24 items, scores range from 0 to 144 points and test-retest reliability is high (*r* = 0.83). The Social Phobia Inventory (SPIN) has 17 items and scores range from 0 to 68 and test-retest reliability is high (*r* = 0.89). The switch from LSAS-SR to SPIN happened when the clinic shifted to the new technical platform.

For panic disorder, the outcome measure was the Panic Disorder Symptom Scale-Self Report (PDSS-SR). PDSS-SR has 7 items and scores range from 0 to 28 points. Test-retest reliability is high (ICC = 0.81) (Houck et al., 2002).

For insomnia the outcome measure was the Insomnia Severity Index (ISI), a seven-item scale ranging from 0 to 28 points (Morin et al., 2011). The scale is reliable and sensitive to change (Bastien et al., 2001).

The Short Health Anxiety Inventory-14 item version (SHAI-14) has, as the name suggests, 14 items (abbreviated from the scales original 64 items). Scores range from 0 to 42. Test-retest reliability is high (*r* = 0.87) (Olatunji et al., 2011).

### 2.3 Statistical analysis

Data artifacts in the waiting time variable from the database were excluded first: If data indicated that the start-of-treatment-measurement was conducted before the screen, this was assumed to be an error. Waiting times above 150 days were also excluded as outliers. This resulted in 497 patients being removed.

A linear regression model was fit for all patients that had MADRS-S as a secondary outcome measure (i.e. patients not in the depression group), with change from screening to start of treatment as the dependent variable and waiting time in days as the independent variable. Change was calculated as start of treatment-score minus screen-score so that more negative scores represent a larger reduction in symptoms since screening.

A second model was fit for primary outcome measures with treatment type as a dummy variable. For models combining primary outcome measures from different treatments the outcomes were scaled (min-max scaling) to range from 0 to 1. This effectively created a percentage of maximum symptom score for each treatment, which are comparable across treatments when aggregated. For the SAD-group this was split up by outcome measure (LSAS-SR and SPIN) as we noticed that combining the measures induced a spurious effect (see supplemantal material).

Exponential models were fit, both for secondary MADRS-S symptoms and for primary outcomes, by transforming symptoms with the logarithmic function. Models with an added quadratic effect of waiting time were also tested.

Finally, a three-way interaction model with start of treatment-score as dependent variable and screening score, waiting time, and treatment group as independent variables was estimated.

Changes in symptoms from screening to start of treatment were tested with paired *t*-tests. Paired Cohen's *d* effect size estimates used average variance (Cumming, 2013).

### 2.4 Simulation and sensitivity power analysis

When planning the project we constructed a simulation to further think through the implication of time-dependent effects (Jaccard and Jacoby, 2010), and to do a power-analysis for our ability to detect such a process. We imagined patient symptoms fluctuare from day to day where many different factors combine to influence whether symptoms increase or decrease relative to the last day. If someone's symptoms depend a lot on how much symptoms they had the day before, that means their fluctuations are more “stable”. If they change a lot randomly each day, the stability is lower. Therefore, each patient is modeled as having a “symptom set-point” which their symptoms fluctuate randomly around with a given stability: the set-point would represent their long term chronic symptom level. In the simulation each patient takes one step per day which regresses towards zero (the set-point) each day by a common stability coefficient, expressed as a correlation coefficient *r*. For example, a simulated patient starting 1 SD away from their set-point, in a simulation where the day-to-day stability is *r* = 0.98, will end up at 1*0.98 + a random normally distributed step with a σ = $\sqrt{1-0.98^{2}}$, ergo: $\mathrm{Scor}e_{t+1}=\mathrm{Scor}e_{t}*r+\sigma$.

Since patients are imagined to self-refer when symptoms are worse than usual for them, the simulation includes this threshold (see Fig. 1). We refer to this as “set-point deviation”, i.e. how far above the set-point does a patient have to deviate before seeking healthcare.

**Fig. 3:**
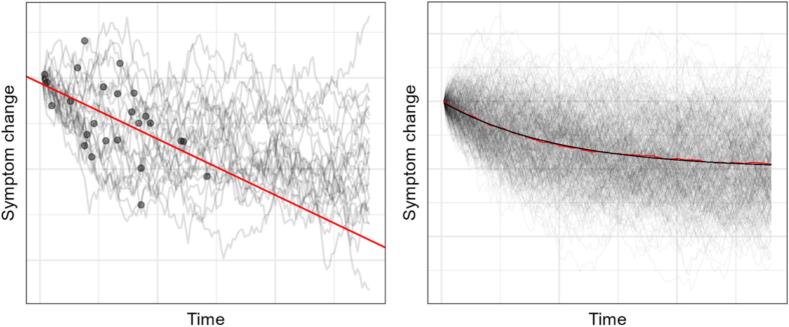
Simulation of symptom trajectories Note. Left: Simulated symptom trajectories of 25 patients changing from screening. Dots represent randomly distributed pre-treatment measurements. Red line is a linear regression based on sampled dots. Right: 400 simulated symptom trajectories. Black line represents mathematically predicted per-day mean. Red line (largely covered by black) represents the mean based on the simulation result.

In the long run these random walks are approximately normally distributed with a standard deviation of one. In large samples of random walks, the per day mean symptom score approaches mean = start deviation*r^*days*^ (see Fig. 3, right). Simulated walks were then sampled once per patient, representing their simulated waiting time between screening and start of treatment (*M* = 29, *SD* = 18). We then ran the simulation 10,000 times each for different values of average set-point deviation, distributed normally around this set-point with *SD* = 0.5, and different levels symptom stability, for *n* = 6700 simulated patients. Prior exploration based on numbers reported in Hedman et al. (2013) has shown that a stability coefficient of ≈ 0.98 would result in the expected drop in symptoms from screening to treatment start, given that the set-point is equal to the end of treatment end point. A day-to-day *r* of 0.98 corresponds to a week-to-week *r* of 0.87. Fig. 3 (left) illustrates an example of the simulation for 25 patients. These trajectories are sampled once for each patient (representing treatment start). As illustrated this symptom fluctuation process would imply a negative relationship between time and symptoms.

The simulation resulted in 100% power for α = 0.05 and *n* = 6700 for all explored levels of stability and set-point deviation (see Supplemental Material). Table 1 below shows resulting *R*^*2*^ for different input parameters. The table can be viewed as a ceiling for how much variance such a symptom fluctuation process could plausibly explain, in different scenarios (given a simple linear regression model).

**Table 1:** R-squared outcomes of simulation for different stability coefficients.

	Stability coefficients (*r*)				
Set-point deviation	0.96	0.97	0.98	0.99	0.995
0.5	1.04	1.11	1.05	0.75	0.44
1	4.03	4.29	4.08	2.93	1.75
1.5	8.57	9.12	8.73	6.37	3.87
2	14.16	15.05	14.49	10.78	6.67

*Note.* Set-point deviations are in standard deviations above the mean which the simulation regresses towards. Stability coefficients represent day-to-day correlation coefficients. Cells represent averaged *R*^*2*^ as percentages from linear models.

### 2.5 Transparency and openness

An analysis-plan was registered at open science framework prior to data analysis (https://osf.io/kah9p). Data were analyzed using R 4.4.0 (R Core Team, 2024). Statistical tests used base R functionality. Addidional packages used were fastDummies (Jacob, 2024), tinytable (Vincent, 2024), ggplot2 (Hadley, 2016), and effectsize (Ben-Shachar et al., 2020). Two major changes in the main analysis happened post-registration in light of our data. First of all regression diagnostics indicated non-normal, fat-tailed, residuals. To address this, the preregistered regression model was supplemented with confidence intervals from a robust regression method using the brms package (Bürkner, 2017). Secondly, combining LSAS-SR and SPIN into a general SAD-index induced a spurious association since the min-maxed scales skew differently, and the eras they were used differ in waiting time (see Supplemental Material). We addressed this by running the analyses without combining them.

Two additional analyses (three-way interaction model and *t*-tests) were added post registration. Finally we investigated the association between screening scores and time as a supplemental analysis. Analysis code has been made publicly available at the open science framework and can be accessed at https://osf.io/td68p/.

Participant data for this study are not available because it is sensitive health care data and we do not have legal permission to make it available even if anonymized.

## 3 Results

Waiting times ranged from 0 to 150 days, with a median of 51 days (*M* = 56.4, *SD* = 32.8). All treatments showed a significant reduction in symptoms from screening to start of treatment, with the exception of SAD when measured with LSAS-SR, which instead showed a small deterioration. When SAD symptoms were measured with SPIN they instead showed a reduction in symptoms like the other treatments. See Table 2 below.

**Table 2:** Within-group effect size for treatment and outcome.

Treatment/Outcome					
	Screeningmean (SD)	Start of treatment mean (SD)	Cohen's *d*_*av*_	Confidence Interval (95%)	Percent reduction due to time⁎
Depression, primary outcome (MADRS-S), *n* = 3452	25.51 (6.28)	23.09 (6.54)	0.38	[0.35, 0.41]	18.7%
Depression, secondary outcome (MADRS-S), *n* = 5292	17.13 (7.93)	15.23 (7.88)	0.25	[0.23, 0.27]	49.7%
Panic disorder (PDSS-SR), *n* = 1795	13.73 (4.99)	11.79 (4.97)	0.36	[0.33, 0.40]	14.5%
Health anxiety (SHAI-14), *n* = 534	30.38 (5.90)	28.62 (6.48)	0.28	[0.23, 0.34]	13.0%
Social anxiety (LSAS-SR), *n* = 1532	71.47 (23.77)	72.18 (23.56)	−0.03	[−0.06, 0.00]	NA⁎
Social anxiety (SPIN), *n* = 367	40.97 (11.10)	39.60 (11.57)	0.14	[0.07, 0.21]	31.0%
Insomnia (ISI), *n* = 659	20.95 (4.11)	18.48 (4.44)	0.58	[0.51, 0.65]	8.1%

*Note.* For some unknown reason the primary outcome data from 111 panic disorder patients and 295 social anxiety patients were lost during data retrieval. MADRS-S ratings was still available for these patients.

⁎ The formula for reduction due to time, explained further below, was not applicable for an increase.

### 3.1 Primary results

**Fig. 4:**
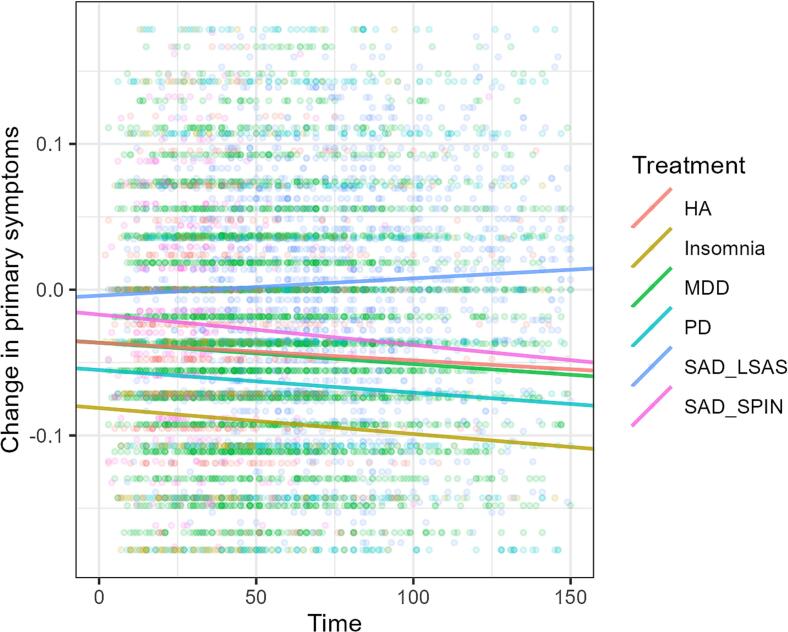
Change in primary symptoms by treatment Note. Plot is zoomed in on the y-axis to more clearly reveal slight differences in slopes. Scores are min-max scaled according to questionnaire range for comparability between treatments. Transparent colored dots and lines represent different treatment subgroups. Abbreviations: HA, health anxiety. MDD, major depressive disorder. PD, panic disorder. SAD, social anxiety disorder.

**Fig. 5:**
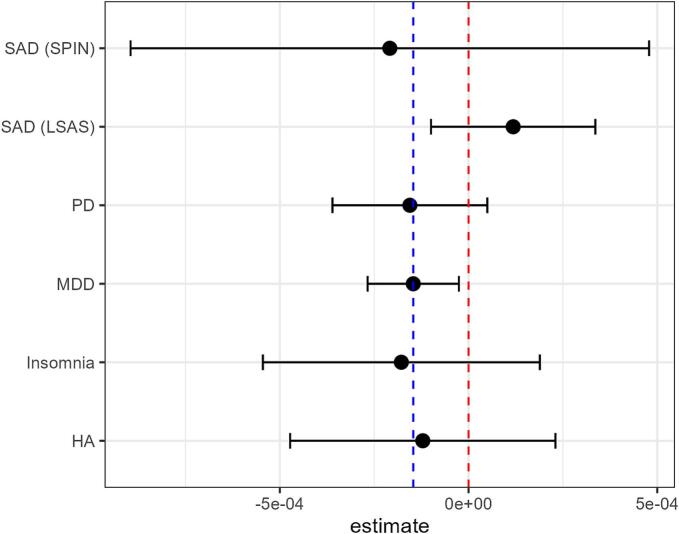
Confidence intervals of time estimates Note. Red dashed line is at zero. Blue dashed line is at the estimate for depression, to clarify its role as reference case in the dummy model.

A linear regression model with primary outcome measures (scaled), waiting time and treatment type as a dummy variable was fit. The model was significant *R2*_*adj*_ *=* 0.05*, F*(12, 8327) = 42.1, *p* < .0001. The relationship between (primary) depression symptoms and time was negative and significant (reference case in the model). The slopes for health anxiety, insomnia and panic disorder did not differ significantly from the reference case (See Table 3 below and Fig. 4), however the SAD when measured with LSAS did. See also Fig. 5 for a more clear view of confidence intervals around the slopes.

**Table 3:** Time dependent change in primary outcome measure.

Variable	Estimate	Std.Error	t.value	p.value	lower.CI	upper.CI
Intercept (Depression)	−0.0364	0.004	−8.922	**0.000**	−0.044	−0.028
Health Anxiety	0.0000	0.010	−0.002	0.998	−0.019	0.019
Insomnia	−0.0448	0.009	−4.807	**0.000**	−0.063	−0.027
Panic Disorder	−0.0187	0.007	−2.622	**0.008**	−0.033	−0.005
Social Anxiety Disorder - LSAS	0.0323	0.009	3.807	**<0.001**	0.016	0.049
Social Anxiety Disorder - SPIN	0.0193	0.013	1.476	0.140	−0.006	0.045
Time	−0.0001	0.0001	−2.375	**0.018**	−0.0003	0.0000
Health Anxiety*Time	0.0000	0.0002	0.141	0.888	−0.0003	0.0004
Insomnia*Time	0.0000	0.0002	−0.169	0.866	−0.0004	0.0003
Panic Disorder*Time	0.0000	0.0001	−0.085	0.932	−0.0002	0.0002
Social Anxiety LSAS*Time	0.0003	0.0001	2.384	**0.017**	0.0000	0.0005
Social Anxiety SPIN*Time	−0.0001	0.0004	−0.177	0.860	−0.0007	0.0006

*Note.* Depression at time difference 0 is reference case in dummy coding. Bolded *p*-values are significant at alpha = 0.05 level. Estimates are min-max scaled according to questionnaire range for comparability between treatments.

Further, we found a significant linear negative relationship between waiting time and symptom change in the treatments where MADRS-S was a secondary symptom measure, *R*^2^_adj_ = 0.01, *F*(2, 5290) = 53.15, intercept = −0.966, *β* = −0.017, *p* < .0001. Patients improved on average 0.12 points on MADRS-S per week waiting time.

#### 3.1.1 Contextualizing primary results using simulation

One way to further make sense of the effect of time is to frame it in relation to the change predicted by the intercept (e.g. change at time = 0). A proportion is calculated with the formula $\beta_{j}\bar{x_{j}}/\left(\alpha_{j}+\beta_{j}\bar{x_{j}}\right)$, for each treatment *j*, where *α* is the intercept, *β* is the slope and $\bar{x}$ is mean waiting time. Note that this means that if the intercept is in another direction than the slope one can end up with percentages above 100% or negative percentages. See rightmost column in Table 3.

This effect too can be contextualized in relation to the expected proportion for different levels of stability and deviation. Table 4. presents the outcomes of 10,000 simulations.

**Table 4:** Percent of change in symptom due to time from simulation.

	Stability coefficient (*r*)				
Set-point deviation	0.96	0.97	0.98	0.99	0.995
0.5	60.6%	68.8%	78.4%	89.9%	96.2%
1	60.5%	68.8%	78.6%	90%	96.5%
1.5	60.5%	68.8%	78.5%	90%	96.4%
2	60.5%	68.9%	78.6%	90%	96.4%

### 3.2 Exploratory analysis

**Fig. 6:**
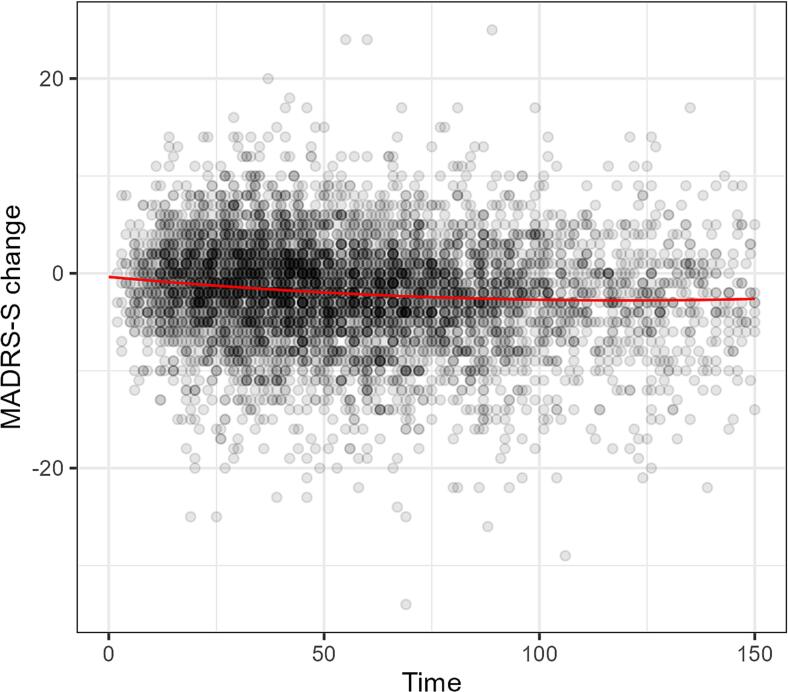
Quadratic model of symptom change in secondary MADRS-S ratings.

Exponential models were fit for waiting time and symptoms change for both primary symptom measures and secondary MADRS ratings. Both exponential models resulted in worse fit (see Supplemental Material). Quadratic models were fit for both primary symptom measures and MADRS. The model of primary outcome was not significantly better, however, the quadratic model for treatments where MADRS-S was a secondary symptom had a significantly better fit *F*(1, 5289) = 7.55, *p* = .006. See Fig. 6 below.

### 3.3 Robust regression

Since regression diagnostics indicated fat-tailed residuals a robust regression using a t-distributed residuals was fit. An exploration of the QQ-plot indicated that residuals were better aligned with a t-distribution with df = 6. A Bayesian t-regression with wide priors was used. Notably the estimate for the time-dependent effect for depression shrunk towards zero, with 95%-CrI only very slightly below zero, *β* = −0.0001 [−0.0002, −0.0000003]. See Table 10 in Supplemental for full result. A Bayesian t-regression for MADRS-S as a secondary outcome was also fit (also df = 6), which yielded similar results to the normal regression, *β* = −0.017 [−0.02, −0.01].

## 4 Discussion

The main finding of this study is that while there is a reduction in symptoms between self-referral and start of treatment, its association with waiting time is very weak. In other words, the drop in symptoms is similar regardless of how long a patient has waited. On the other hand, the total reductions in symptoms are of a size that we consider to be highly relevant for the estimation of effects in single-group psychotherapy trials.

The time-dependent reductions in depressive symptoms, are consistent with the hypothesis that individuals seek treatment when their fluctuating symptoms are worse than usual, and then improve as symptoms statistically regress towards “as bad as usual”. Regarding other primary symptoms the evidence is more ambiguous, and should not be interpreted as strong evidence for a time-dependent effect: In our pre-registered model most min-max scaled estimates land closer to the depression estimate than to zero, but their confidence intervals also overlap zero (Fig. 5).Finally, the estimate for primary depressive symptoms shrunk towards zero when using robust regression. Although the 95% credible interval did not overlap zero, this sensitivity to modeling decisions should warrant some caution when interpreting the effect.

When planning this project we developed a simulation of symptom fluctuation to explore a theoretically plausible data-generating process of a time-dependent effect. Since that simulation implied non-linear trajectories, we also explored non-linear models. For comorbid depressive symptoms a quadradic fit was superior. We note that the curved appearance of this diminishing effect from symptom fluctuation is similar to that predicted from our simulated data. Though the data generating process in our simulation is not itself quadratic, quadratic models tend to give a superior fit to simulated data compared to linear models. However, this should not be taken as strong support for any specific mechanism.

The effect of waiting time is small, and we only find clear evidence for it for depressive symptoms. It is most evident for comorbid depressive symptoms, but even there the model explains only 1% of the variance. To fully contextualize this effect size however it is helpful to look at Table 2 from the simulated power-analysis: Since fluctuating symptoms are fundamentally a random process, even in an idealized scenario it would be unreasonable to expect a perfectly predictable rate of spontaneous improvement. The simulation can be seen as exemplifying a potential upper limit of the effect in a scenario where random symptom fluctuation is the one dominant process that explains the reduction in symptom scores (given assumptions of how those fluctuations might behave).

Even so, we should emphasize that the reduction in scores that we see is mainly time-independent. Only a small proportion of the total reduction is time dependent. It is potentially interesting to note that it is comparatively stronger for comorbid depressive symptom (49.7% in the linear model). One possible interpretation of this contrast is that there is a (weak) tendency for patients to seek help for their more stable syndromes at a time when their comorbid mood symptoms, or general distress, are worse than usual, although this is highly speculative.

While the causes of these effects, both time-dependent and time-independent are not identifiable from the data, we believe the descriptive results are important in and of themselves. Spontaneous remission in psychiatric symptoms is often discussed on a larger time scale (e.g. in the context of passive wait-list control groups (Cuijpers et al., 2021; Whiteford et al., 2013) but this study is, to our knowledge, the first confirmation of the phenomenon for patients waiting to start treatment. Given the small effects we found, and that small effects are expected according to our simulation, it is possible that some earlier studies or exploratory data analyses have been underpowered to detect the phenomenon (e.g. Menéndez et al., 2025).

### 4.1 Implications

Controlled trials and evaluations of psychological treatments in regular care differ in how and when they measure outcomes. In many mental health services symptom measurements are administered at the first visit as part of a wider assessment for treatment eligibility. A researcher could plausibly assume that if this measurement happens shortly before the start of treatment, that implies it is equivalent to a measurement *at* the start of treatment. However, in our data most of the change in primary symptoms between screening and start of treatment had nothing to do with the time elapsed between those two timepoints. Whether this change happens right after screening, after the assessment, or right at the point of starting the treatment is unknown, but for the purpose of measurement it can be regarded as “immediate”. This means that it is highly important for research and clinical evaluations that rely on within-group analysis be mindful and transparent about what type of measurements are being used as the starting point for analysis of treatment effects. Failing to account for this pre-treatment improvement (in the present data as high as *d*_*av*_ = 0.58, for insomnia) can otherwise create inflated estimates of treatment effects where non-specific pre-treatment improvements are mixed with improvements that occur during the treatment period.

Based on our present data, the converse mistake where researchers feel confident in the validity of their pre-treatment measurement, but are inattentive to the aspect of waiting time, would be less detrimental and mostly relevant for comorbid depressive symptoms. Even so, the present study should not be interpreted as if waiting times or treatment lengths are unimportant for psychotherapy research. For example, the problem with combining pre-post treatment effects from treatments of different lengths in meta-analyses, has previously been emphasized by Cuijpers et al. (2017).

Our recommendation is that researchers thoroughly report the context in which measurements happen, as well as report waiting times before treatment starts. If separate screening and start-of-treatment measures are collected, both should be reported. We regard using assessments that determine eligibility for treatment as more problematic than start-of-treatment measurements, as that is bound to introduce a regression towards the mean effect.

### 4.2 Limitations

This study has several limitations. Firstly, this is an observational study in a routine care context with no experimental intervention and as such the data collection was not made with this analysis in mind. Because of this, we have no formal clinical assessment of improvement at the start of treatment as is often the case when, for instance, examining test-retest reliability. It is therefore difficult to separate the effect of measurement error from the general effect on symptoms. However, our primary analysis, detecting the effect of waiting time, is not affected by this limitation.

Part of the total reduction in symptom scores from screening to start of treatment may be due to patients below a certain threshold being excluded from treatment. However, we believe this effect is limited somewhat by the fact that the Internet Psychiatry Clinic has no formal inclusion criteria based on screening symptom scores. Whether a patient starts treatment is determined by the assessing clinician.

There are multiple potential confounders that undermine a causal interpretation of the time-dependent effect. Firstly, more severe patients may be prioritized for earlier assessments. However, we only found a significant association in that direction for panic disorder (see Supplemental Material). For comorbid depressive symptoms we, surprisingly, saw a relationship in the other direction. Furthermore, our estimation of the time-dependent effect depends on fluctuations in waiting time at the clinic, but it is possible these fluctuations are confounded with unknown cohort effects, or with other changes in the clinic that might affect ratings. For example, meeting a psychologist or doctor for assessment at a time where waiting times are long at the clinic may give patients a different impression of overall treatment/clinic credibility, compared to when waiting times are short. A final confounder is that some patients may choose to wait longer, for whatever reason. If, for example, feeling better due to the assessment visit reduces motivation to start treatment, this could induce a spurious time-dependent effect due to factors we classify as time-independent. Other individual-level confounders could instead mask the effect.

The design of the study precludes conclusion regarding specific causes. Future research could mitigate this by either explicitly randomizing waiting time or including appropriate control variables.

Participants at the clinic tend to be Swedish citizens with a majority being highly educated. It is unknown if the fact that the patients are screening for, and later starting, internet-delivered, as opposed to face-to-face, treatment has any bearing on the results. The treatment itself has not had a chance to affect the outcome, but the context could potentially have an effect on self-selection into the sample, limiting generalizability to patients in other contexts. These results should be replicated in other settings and demographics to explore further.

### 4.3 Conclusion

Average reductions in symptoms between screening or referral and the start of treatment is a common phenomenon. A prima facie plausible hypothesis is that this change is due to patients seeking treatment when their fluctuating symptoms are worse than usual. The present study is the first to show such a time-dependent effect in a clinical context. However, the effects were small and we only found clear evidence for it for depressive symptoms. Instead, most of the reduction appears irrespective of waiting time. This may imply that the context and processes around the measurements tend to matter more than time elapsed between measurements. Not accounting for this may lead to an overestimation of clinical effectiveness.

## Funding

This work was supported by Region Stockholm via the Center for Innovative Medicine – CIMED (FoUI-963433), L.J. Boëthius stiftelse and by Stiftelsen Bror Gadelius Minnesfond.

## Declaration of competing interest

The authors declare that they have no known competing financial interests or personal relationships that could have appeared to influence the work reported in this paper.

## Appendix A Supplementary data

Supplementary material Image 1
