# Supplementary material for: Not just spontaneous remission: Time-dependent and independent effects in pre-intervention symptom reduction
Source: Internet Interv. 2026 Feb 27;44:100926. doi: 10.1016/j.invent.2026.100926 (PMC12969135; doi:10.1016/j.invent.2026.100926)
Supplement: Supplementary file 1 — Supplementary material [file mmc1.docx]

# **Supplemental Material**

An additional prediction from the symptom fluctuation hypothesis is that the relationship between screening scores and start-of-treatment scores should get weaker as waiting time increases. We thus explored whether waiting-time predicted symptom change differently in a three-way interaction model with start-of-treatment-score as dependent variable and screening-score, waiting time, and treatment type as independent variables. Depression is still the reference case. Selected results are described below:

Depressive symptoms at screening were significantly associated with symptoms at start-of-treatment, *β* = 0.76, [0.70, 0.83], *t* = 24.17, *p* < .0001, and this association was significantly stronger for health anxiety, *β* = 0.20, [0.06, 0.34], *t* = 2.88, *p* = .004. Start of treatment scores for depression got lower as time increase, *β* = -0.0002 [-0.0003, -0.0001], *t* = 3.20, *p* = .014. There was a significant interaction in the depression group between screening-score and waiting time, *β* = -0.0017 [-0.0026, -0.0007], *t* = -3.44, *p* = .0006, meaning that as waiting time increases the relationship between screening scores and pre-scores get weaker. This interaction was not significantly different for panic disorder, health anxiety and insomnia. However, the interaction was significantly different for SAD measured by LSAS, *β* = 0.002 [0.001, 0.003], *t* = 2.63, *p* = .009.

**Table 6**

*Three-way Interaction Model Predicting Start of Treatment Symptoms*

|  | Estimate | Std.Error | t-value | p-value | Lower CI | Upper CI |
| --- | --- | --- | --- | --- | --- | --- |
| Intercept (MDD) | 0.4379 | 0.0038 | 116.1247 | **0.0000** | 0.4305 | 0.4453 |
| Screening | 0.7633 | 0.0316 | 24.1717 | **0.0000** | 0.7014 | 0.8253 |
| Time | -0.0002 | 0.0001 | -3.1986 | **0.0014** | -0.0003 | -0.0001 |
| HA | 0.2500 | 0.0092 | 27.2476 | **0.0000** | 0.2321 | 0.2680 |
| Insomnia | 0.2374 | 0.0086 | 27.5028 | **0.0000** | 0.2204 | 0.2543 |
| PD | 0.0101 | 0.0066 | 1.5309 | 0.1258 | -0.0028 | 0.0231 |
| SAD - LSAS | 0.0569 | 0.0078 | 7.2524 | **0.0000** | 0.0415 | 0.0722 |
| SAD - SPIN | 0.1520 | 0.0121 | 12.5490 | **0.0000** | 0.1282 | 0.1757 |
| Screening*Time | -0.0017 | 0.0005 | -3.4421 | **0.0006** | -0.0026 | -0.0007 |
| Screening*HA | 0.2003 | 0.0696 | 2.8796 | **0.0040** | 0.0640 | 0.3367 |
| Screening*Insomnia | -0.0754 | 0.0621 | -1.2148 | 0.2245 | -0.1970 | 0.0463 |
| Screening*PD | -0.0448 | 0.0439 | -1.0210 | 0.3073 | -0.1309 | 0.0412 |
| Screening*SAD - LSAS | 0.0702 | 0.0513 | 1.3666 | 0.1718 | -0.0305 | 0.1708 |
| Screening*SAD - SPIN | 0.0864 | 0.0745 | 1.1598 | 0.2461 | -0.0596 | 0.2324 |
| Time*HA | 0.0000 | 0.0002 | 0.1259 | 0.8998 | -0.0003 | 0.0003 |
| Time*Insomnia | -0.0002 | 0.0002 | -1.2041 | 0.2286 | -0.0005 | 0.0001 |
| Time*PD | -0.0002 | 0.0001 | -2.0306 | **0.0423** | -0.0004 | 0.0000 |
| Time*SAD - LSAS | 0.0003 | 0.0001 | 2.5945 | **0.0095** | 0.0001 | 0.0005 |
| Time*SAD - SPIN | -0.0002 | 0.0003 | -0.4724 | 0.6367 | -0.0008 | 0.0005 |
| Screening*Time*HA | -0.0007 | 0.0013 | -0.5200 | 0.6031 | -0.0031 | 0.0018 |
| Screening*Time*Insomnia | 0.0005 | 0.0012 | 0.4289 | 0.6680 | -0.0018 | 0.0028 |
| Screening*Time*PD | 0.0004 | 0.0007 | 0.5493 | 0.5828 | -0.0009 | 0.0017 |
| Screening*Time*SAD - LSAS | 0.0018 | 0.0007 | 2.6253 | **0.0087** | 0.0005 | 0.0032 |
| Screening*Time*SAD - SPIN | 0.0003 | 0.0019 | 0.1505 | 0.8804 | -0.0035 | 0.0041 |

*Note.* Depression at time difference 0 is reference case in dummy coding. Bolded p-values are significant at alpha = .05 level. Estimates are min-max scaled according to questionnaire range for comparability between treatments. Screening has been mean centered per treatment.

**Table 7**

*Result of Simulated Power Analysis (n = 6700)*

|  |  | Stability coefficients (*r*) | | | | |
| --- | --- | --- | --- | --- | --- | --- |
| Set-point deviation |  | **0.96** | **0.97** | **0.98** | **0.99** | **0.995** |
| **0.5** |  | 1 | 1 | 1 | 1 | 1 |
| **1** |  | 1 | 1 | 1 | 1 | 1 |
| **1.5** |  | 1 | 1 | 1 | 1 | 1 |
| **2** |  | 1 | 1 | 1 | 1 | 1 |

*Note.* Cells represent proportion significant/total from simulations. Theoretically, statistical power is never 100% but all simulations were significant.

**Analysis of SPIN contra LSAS scores**

In our post-hoc analysis we noted that the min-maxed scores on SPIN and LSAS skew very differently at screening. See histograms below:


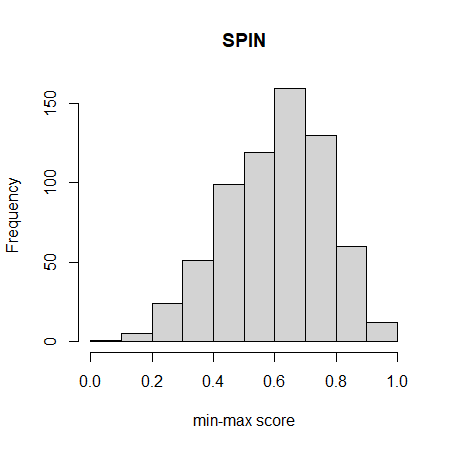

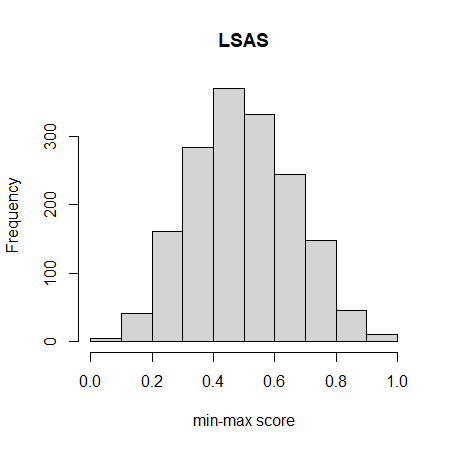


Our interpretation is that a similar symptom level is differently far from the max-score of the questionnaires. This in combination with LSAS-scores being used during a time where waiting time was longer (median = 66) compared to when SPIN was used (median = 32) is seen as a sufficient explanation for the observed positive trend in the aggregated SAD-scores. This motivated us to split up the scores in the primary analysis.

**Exponential and quadratic models:**

The quadratic model of primary symptoms was not significantly better fit than the linear model, *F*(5, 8324) = 0.689, *p* = 0.632. Results are presented in the table below:

**Table 8**

*Quadratic model*

| Var | Estimate | Std.Error | t.value | p.value | Lower.CI | upper.CI |
| --- | --- | --- | --- | --- | --- | --- |
| MDD (Intercept) | -0.045 | 0.002 | -22.83 | **0.000** | -0.048 | -0.041 |
| HA | 0.001 | 0.006 | 0.194 | 0.846 | -0.010 | 0.012 |
| Insomnia | -0.046 | 0.006 | -8.202 | **0.000** | -0.057 | -0.035 |
| PD | -0.019 | 0.003 | -5.554 | **0.000** | -0.025 | -0.012 |
| SAD | 0.042 | 0.003 | 12.384 | **0.000** | 0.035 | 0.048 |
| poly(Time, 2)1 | -0.463 | 0.193 | -2.398 | **0.016** | -0.842 | -0.084 |
| poly(Time, 2)2 | 0.076 | 0.198 | 0.385 | 0.700 | -0.312 | 0.465 |
| HA*poly(Time, 2)1 | 0.199 | 0.572 | 0.348 | 0.728 | -0.922 | 1.320 |
| HA*poly(Time, 2)2 | 0.444 | 0.573 | 0.775 | 0.439 | -0.680 | 1.568 |
| Insomnia*poly(Time, 2)1 | 0.055 | 0.633 | 0.088 | 0.930 | -1.185 | 1.296 |
| Insomnia*poly(Time, 2)2 | 0.256 | 0.584 | 0.438 | 0.661 | -0.888 | 1.399 |
| PD*poly(Time, 2)1 | -0.089 | 0.331 | -0.269 | 0.788 | -0.738 | 0.559 |
| PD*poly(Time, 2)2 | 0.287 | 0.334 | 0.859 | 0.391 | -0.368 | 0.941 |
| SAD*poly(Time, 2)1 | 1.172 | 0.320 | 3.661 | **0.000** | 0.544 | 1.799 |
| SAD*poly(Time, 2)2 | 0.024 | 0.320 | 0.074 | 0.941 | -0.603 | 0.650 |

**Table 9**

*Exponential model.*

| Var | Estimate | Std.Error | t.value | p.value | lower.CI | upper.CI |
| --- | --- | --- | --- | --- | --- | --- |
| MDD (Intercept) | -0.293 | 0.007 | -40.713 | **0.000** | -0.307 | -0.278 |
| HA | -0.002 | 0.017 | -0.098 | 0.922 | -0.036 | 0.033 |
| Insomnia | -0.072 | 0.016 | -4.397 | **0.000** | -0.104 | -0.040 |
| PD | -0.042 | 0.013 | -3.366 | **0.001** | -0.067 | -0.018 |
| SAD | 0.022 | 0.012 | 1.809 | 0.070 | -0.002 | 0.047 |
| Time | 0.000 | 0.000 | -2.862 | **0.004** | -0.001 | 0.000 |
| HA*Time | 0.000 | 0.000 | 0.228 | 0.820 | -0.001 | 0.001 |
| Insomnia*Time | 0.000 | 0.000 | -1.402 | 0.161 | -0.001 | 0.000 |
| PD*Time | 0.000 | 0.000 | 0.330 | 0.741 | 0.000 | 0.000 |
| SAD*Time | 0.001 | 0.000 | 3.574 | **0.000** | 0.000 | 0.001 |

*Note.* Estimates of symptom change is now the natural logarithm of the min-maxed scale.

**Bayesian robust t-regression:**

The regression uses t-distributed residuals (df = 6) instead of normal residuals. QQ-plots for residuals from initial regression models are presented below the table. For comparability to our frequentist linear models we used wide priors: Normal(0,10) for both intercepts and slopes, and Exponential(2) for sigma. The mode was run with 4 chains and 2000 iterations. R-values and traceplots indicated good convergence for all parameters.

**Table 10**

*Bayesian robust t-regression*

|  | **Estimate** | **Std. Error** | **Lower CrI** | **Upper CrI** | **Rhat** | **Bulk ESS** |
| --- | --- | --- | --- | --- | --- | --- |
| MDD Intercept | -0.0363275 | 0.0035909 | -0.0435137 | -0.02925029 | 1 | 2512 |
| HA | 0.0004194 | 0.0086044 | -0.0164319 | 0.01715196 | 1 | 2703 |
| Insomnia | -0.045606 | 0.008867 | -0.0629533 | -0.02850402 | 1 | 2567 |
| PD | -0.0129883 | 0.0070049 | -0.0269375 | 0.00094483 | 1 | 2747 |
| SAD - LSAS | 0.0327017 | 0.0073803 | 0.0178884 | 0.04763597 | 1 | 2721 |
| SAD – SPIN | 0.019404 | 0.0115786 | -0.0031065 | 0.04174348 | 1 | 2830 |
| Time | -0.0001084 | 0.0000548 | -0.0002184 | -0.00000032 | 1 | 2962 |
| HA*Time | 0.0000149 | 0.0001591 | -0.0002956 | 0.00033305 | 1 | 3334 |
| Insomnia*Time | 0.0001328 | 0.0001762 | -0.0001987 | 0.00048128 | 1 | 3153 |
| PD*Time | -0.0000642 | 0.0001037 | -0.0002718 | 0.00014245 | 1 | 3008 |
| SAD-LSAS*Time | 0.0002108 | 0.0000979 | 0.0000162 | 0.00040311 | 1 | 3017 |
| SAD-SPIN*Time | -0.0000567 | 0.0003194 | -0.0006844 | 0.0005581 | 1 | 3136 |

*Note.* Credible intervals (CrI) are 95%.

**QQ-plots:**

*Primary outcomes model, normal distribution residuals*


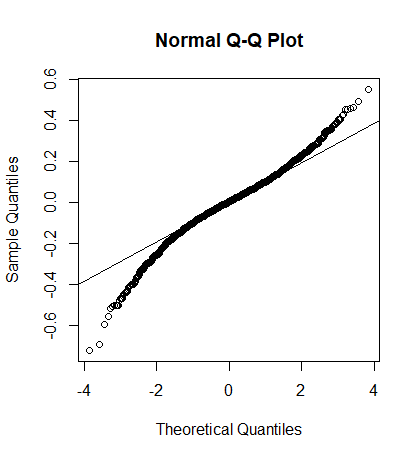


*Primary outcomes model, t-distribution residuals*


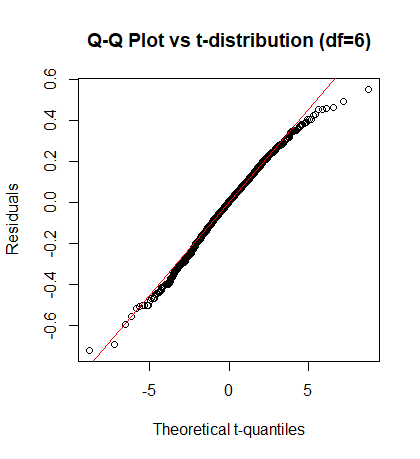


*Secondary MADRS model, normal residuals*


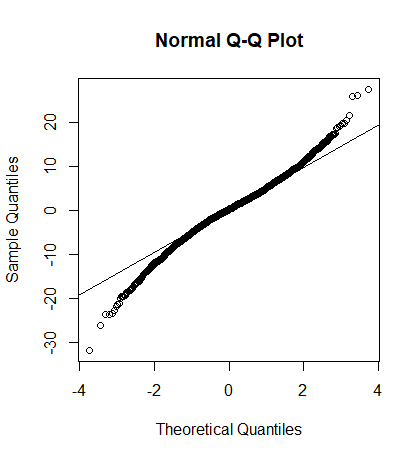


*Secondary MADRS model, t-distribution residuals*

*
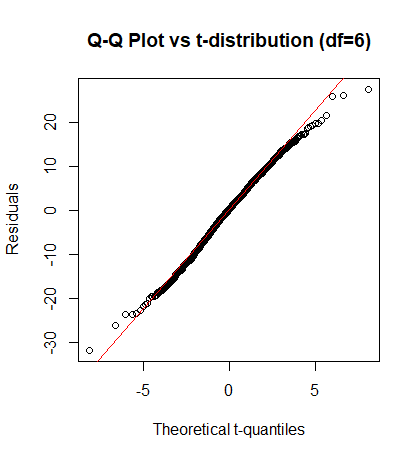
*

**Total model fit change:**

One way to describe an overall influence of waiting time is to estimate how much adding the time variable to the model increases overall model fit. To do this first a model with primary outcome measures and treatment type as a dummy variable was fit, without the time variable. This model was then compared to a model where waiting time was also added as an interaction. The full model resulted in a non-significantly improved fit, *F*(6, 8327) = 2.09, *p* = .051, intercepts model *R^2^_adj_* = .0507 vs full model *R^2^_adj_* = .0515.

**Association between screening score and time:**

Patients that have more severe symptoms could potentially be prioritized for assessments at the clinic, or have a tendency to log on quicker after the treatment is available. Because of this risk, it is helpful to look at the raw association between screening score and waiting time. The association was insignificant for all treatments except panic disorder (table 11 below). For patients with panic disorder shorter waiting time was associated with higher screening scores. There was also a weak but significant association for comorbid MADRS-S score: *R^2^_adj_* = 0.001, *F*(2, 5412) = 4.431, *β* = 0.0001, *p* = 0.035. This association was reversed, longer waiting time for patients with higher screening scores.

**Table 11**

*Screening score regressed on time*

| Var | Estimate | Std.Error | t.value | p.value |
| --- | --- | --- | --- | --- |
| MDD (Intercept) | 0.00766 | 0.00528 | 1.44952 | 0.14723 |
| Time | -0.00013 | 0.00008 | -1.64663 | 0.09967 |
| HA | 0.00829 | 0.01283 | 0.64597 | 0.51832 |
| Insomnia | 0.01491 | 0.01208 | 1.23432 | 0.21712 |
| PD | 0.03072 | 0.00924 | 3.32524 | **0.00089** |
| SAD – LSAS | 0.00669 | 0.00996 | 0.67178 | 0.50174 |
| SAD – SPIN | -0.00570 | 0.01210 | -0.47134 | 0.63741 |
| Time*HA | -0.00022 | 0.00023 | -0.96138 | 0.33639 |
| Time*Insomnia | -0.00043 | 0.00024 | -1.76814 | 0.07707 |
| Time*PD | -0.00049 | 0.00014 | -3.62466 | **0.00029** |
| Time*SAD – LSAS | -0.00007 | 0.00013 | -0.53548 | 0.59233 |
| Time*SAD – SPIN | 0.00008 | 0.00026 | 0.29810 | 0.76564 |

*Note.* Depression is reference case in dummy coding. Bolded p-values are significant at alpha = .05 level. Estimates are min-max scaled according to questionnaire range for comparability between treatments.
